# An Infrared and Visible Image Fusion Network Based on Res2Net and Multiscale Transformer

**DOI:** 10.3390/s25030791

**Published:** 2025-01-28

**Authors:** Binxi Tan, Bin Yang

**Affiliations:** College of Electrical Engineering, University of South China, Hengyang 421001, China; tanbinxi0508@163.com

**Keywords:** image fusion, Transformer, multiscale features, infrared image, deep learning

## Abstract

The aim of infrared and visible image fusion is to produce a composite image that can highlight the infrared targets and maintain plentiful detailed textures simultaneously. Despite the promising fusion performance of current deep-learning-based algorithms, most fusion algorithms highly depend on convolution operations, which limits their capability to represent long-range contextual information. To overcome this challenge, we design a novel infrared and visible image fusion network based on Res2Net and multiscale Transformer, called RMTFuse. Specifically, we devise a local feature extraction module based on Res2Net (LFE-RN) in which dense connections are adopted to reuse the information that might be lost in convolution operation and a global feature extraction module based on multiscale Transformer (GFE-MT) which is composed of a Transformer module and a global feature integration module (GFIM). The Transformer module extracts the coarse-to-fine semantic features of the source images, while GFIM is used to further aggregate the hierarchical features to strengthen contextual feature representations. Furthermore, we employ the pre-trained VGG-16 network to compute the loss of features with different depths. Massive experiments on mainstream datasets indicate that RMTFuse is superior to the state-of-the-art methods in both subjective and objective assessments.

## 1. Introduction

With images captured by one-modality sensors, it is difficult to comprehensively and efficiently characterize the imaging scene on account of theoretical and technological limitations [1]. The visible sensor describes the scene texture details by reflective visible light, but it is vulnerable to various environmental interference like scene brightness. By comparison, the infrared sensor highlights the radiative properties of salient targets through thermal radiation, but it ignores details and is also susceptible to noise. Visible and infrared image fusion can integrate complementary features from the two types of images, resulting in an informative fusion result and thereby promoting numerous visual tasks [2], such as semantic segmentation [3], object tracking as well as detection [4,5,6], and biometric recognition [7].

In the last few decades, researchers have put forward a large number of fusion algorithms for infrared and visible images. Generally, they are divided into two main categories: traditional image fusion methods and deep-learning-based fusion models. Traditional image fusion methods are concerned with extracting important features from the original images. The method on the basis of multiscale transformation [8,9] is a common traditional method for image fusion. It first extracts multiscale features from original images and subsequently merges these features using hand-crafted fusion rules. To reconstruct the fusion image, the inverse multiscale transformation is finally adopted. The main challenge of this type of fusion method lies in effectively extracting features from the original images and formulating appropriate rules to obtain an informative fusion image. Aside from multiscale transformation, other traditional image fusion algorithms include subspace-based [10], sparse-representation-based [11], optimization-based [12], hybrid methods [13], and others [14]. However, a great hindrance is present in the advancement of traditional image fusion techniques. For one thing, such handcrafted fusion strategies and rules cannot fully leverage the complementary features of two different modalities. For another, these traditional methods are not suitable for integration in complicated scenes as a result of the limitation of representation ability.

In the past decade, deep learning theories have been deployed in diverse computer vision challenges successfully, thus promoting the implementation of image fusion approaches based on deep learning theories. Existing deep-learning-based methods are roughly categorized into three types, namely convolutional neural network (CNN)-based methods [15,16], autoencoder (AE)-based methods [17,18], and generative adversarial network (GAN)-based methods [19,20]. Owing to their powerful capability in extracting features, CNN-based fusion methods effectively address the manual design issues presented in traditional methods by performing integration strategies and activity level measurements holistically through convolution operations. They concentrate on the way to devise excellent network frameworks and formulate appropriate loss functions to perform feature extraction, fusion, and reconstruction, ultimately achieving distinctive fused results. Unlike CNN-based algorithms, the fusion methods based on AE train both the encoder and decoder to achieve the extraction and reconstruction of complementary features, whereas the process of merging these obtained features is accomplished through carefully designed fusion rules. In contrast, the methods based on GAN introduce the generative adversarial mechanism into the fusion network, thereby constantly optimizing the generated results and forcing them to approximate the ideal probability distribution with non-supervision.

With powerful feature extraction and generalization abilities, deep-learning-based fusion methods have made considerable progress in the image fusion domain, far surpassing traditional methods. Unfortunately, these deep learning methods above tend to lose global features as they rely on the CNN to execute particular operations during the phase of feature extraction [21]. The CNN indeed has a good local feature extraction ability and generalization ability, but it struggles to effectively capture long-range dependencies in images owing to the limited receptive field. In contrast, Transformer performs well in modeling the long-range dependencies of images. With this motivation, we develop an effective fusion network for infrared and visible images that successfully integrates the CNN and vision Transformer. The fusion network can maintain the merit of the CNN as well as improve the long-range dependencies of input images, thus enhancing fusion quality. Specifically, a local feature extraction module based on Res2Net (LFE-RN) is devised to fully utilize the multiscale features of a local neighborhood. In order to avoid weakening some beneficial information for fusion, we apply dense connections in LFE-RN. Moreover, we devise a global feature extraction module based on multiscale Transformer (GFE-MT) for further preserving global context semantic information.

The primary contributions in this paper can be characterized as follows:

- We put forward a novel and efficient network that combines CNN and Transformer for the fusion of infrared and visible images. The proposed network performs well in integrating complementary information by effectively utilizing both local and global features of source images.
- We devise a densely shaped LFE-RN to effectively exploit local features and reuse information that could be lost during the feature extraction operation. A Transformer module and global feature integration module (GFIM) are devised in GFE-MT to further preserve global contextual information.
- A targeted perceptual loss function is devised to retain the high similarity between source images and the fused result.
- Extensive experiments on two dominant datasets, i.e., TNO and RoadScene, illustrate that our method surpasses other state-of-the-art image fusion methods in terms of both subjective effects and objective evaluations.

The remainder of this paper is organized in the following manner: In Section 2, a brief overview of relevant research on image fusion is provided. Section 3 introduces our RMTFuse in detail. In Section 4, we carry out an ablation study to validate the impacts of each module, as well as illustrate the outstanding performance of RMTFuse compared to other methods. Finally, some concluding remarks on our work are presented in Section 5.

## 2. Related Work

In this section, we first review the recent advances in deep-learning-based image fusion methods, followed by a detailed description of Res2Net and vision Transformer.

### 2.1. Deep-Learning-Based Image Fusion Methods

#### 2.1.1. CNN-Based Fusion Methods

Liu et al. [22] were pioneers in utilizing CNNs for image fusion tasks. In their research, a CNN was employed to calculate a binary map to obtain fused multi-focus images. Subsequently, Liu et al. [23] introduced a Siamese CNN into the network to acquire weight maps from input images to settle the problem of integrating infrared and visible images. They broke input images into pyramids and conducted fusion in a multiscale manner. In [16], Zhang et al. converted the problem of unified image fusion to maintain the radio of gradient and intensity in the source images. They also proposed a generic loss function applicable to different fusion tasks. Xu et al. [24] developed a unified fusion model that estimated the significance of different source images in an adaptive way. In order to adequately exploit valuable information from source images, Guo et al. [25] evaluated the weight score matrix of input images to identify the respective contributions of each image and introduced a masking strategy into their loss function. To better facilitate the downstream tasks during image fusion, a semantic-aware fusion framework was designed in [26], namely SeAFusion.

#### 2.1.2. AE-Based Fusion Methods

Benefiting from the development of deep learning, some researchers have put forward the AE-based image fusion algorithms. In order to accomplish feature extraction and image reconstruction, most of them pre-train an autoencoder on large-scale datasets. Subsequently, an appropriate fusion rule is devised to perform the feature fusion task. A famous and pioneering AE-based method for the integration of infrared and visible images was DenseFuse [27]. It adopted a dense block for extracting image features and selected an additive strategy or l1-norm strategy to achieve feature fusion. Furthermore, Li et al. [28] brought nest connections into the network to extract multiscale features. Nevertheless, they all applied manual fusion strategies to integrate features, weakening the performance of pre-trained fusion model. To tackle this problem, they later presented an end-to-end fusion framework [17], namely RFN-Nest, where feature fusion is performed by a learnable residual fusion framework. Considering that multiscale characteristics greatly affect the performance of fused images, Wang et al. [29] developed a multiscale encoder–decoder fusion network based on Res2Net and double nonlocal attention models to retain as much significant information as possible. More recently, a multilevel dual-branch attention network was proposed in MDAN [18] to effectively reduce the information loss in the feature extraction process, so as to obtain an information-rich fused image.

#### 2.1.3. GAN-Based Fusion Methods

A GAN can effectively evaluate probability distributions in an unsupervised way, which makes it a good choice for image fusion tasks. In 2019, Ma et al. [20] firstly introduced a GAN into the image fusion field, casting the fusion task as an antagonistic game between a generator and a discriminator. Unfortunately, the obtained result closely resembled a sharpened infrared image as there is only one discriminator. Subsequently, they further introduced dual discriminators into the GAN-based image fusion to achieve fusion balance [30]. In GANMcC [31], the image fusion issue is converted into the multi-classification constraint, and the obtained fused result is more balanced. In 2022, a target-aware adversarial model was devised in TarDAL [32] that effectively combined image fusion and follow-up detection tasks. More recently, in MAGAN [33], multi-attention mechanisms were introduced into a generator and two discriminators to fuse images selectively. However, GANs are hard to optimize, and the generated images may be biased towards either of the input images.

Although the above deep-learning-based fusion methods have achieved satisfactory results, there are still some shortcomings that need to be addressed. On the one hand, most of these fusion methods only employ the feature maps of the final layer and are unable to preserve features across different scales, which may weaken the useful information for the image fusion task. On the other hand, these methods usually rely on convolution operations, which fail to fully capture global significant features because of the limited receptive fields. As a result, the Res2Net block and multiscale vision Transformer are introduced into the proposed model to extract the multiscale local and global features, improving the quality of fused images.

### 2.2. Res2Net

**Figure 1:**
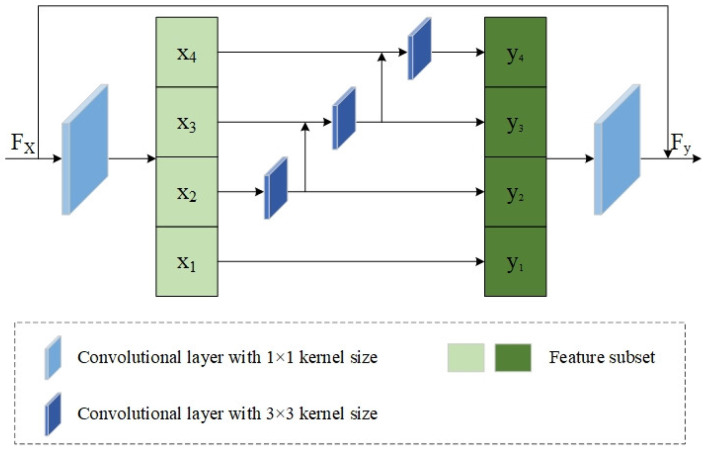
Architecture of Res2Net.The feature information of different depths is obtained by a multiscale residual connection module.

In [34], a new multiscale backbone module called Res2Net was proposed to strengthen the multiscale expression ability of a CNN. It constructs hierarchical residual connections to represent multiscale features at a fine-grained level. Figure 1 shows the detailed architecture of Res2Net. A 1 × 1 convolutional layer is first adopted for adjusting the channel numbers of input feature maps to facilitate subsequent processing. The gained feature maps are then equally divided into *s* feature subsets that have the same spatial size, where *s* is the scale control parameter. Afterward, with the exception of the first one, every subset undergoes the operation with a corresponding convolution of 3 × 3 and is integrated into the following feature subset. Then, all of these acquired features are fed into another 1 × 1 convolution and connected with primitive feature information, thereby obtaining an output result that has a larger receptive field.

In this paper, the Res2Net is viewed as a convolution block and introduced into the fusion framework for fine-grained multiscale feature extraction. In addition, dense connections are employed to achieve feature reuse and strengthen information propagation. The scale control parameter *s* is set to 4 in subsequent experiments.

### 2.3. Vision Transformer

In 2017, the Transformer architecture was first proposed [35] and employed in natural language processing (NLP) tasks, such as word prediction and sentiment categorization. Motivated by the success of Transformer in NLP, the vision Transformer (ViT) structure was put forward by Dosovitskiy et al. [36] to perform image classification tasks. They split the input images into 16 × 16 patches, which were then fed into a standard Transformer. Given its powerful ability for long-range modeling, Transformer has excelled in various visual tasks, which include target detection, semantic segmentation, image restoration, etc. This contributed to the application of Transformer-based networks in the domain of image fusion. In 2021, VS et al. [37] designed a novel fusion strategy based on spatial CNN and Transformer branches to fuse local and global semantic information. Ma et al. [38] devised a unified fusion network on the basis of Swin Transformer. Specifically, the Transformer was utilized to represent the long dependencies within the same domain as well as across domains for better complementary information integration. In addition, Tang et al. [39] proposed DATFuse, in which a dual attention residual structure and Transformer were combined to achieve better fusion performance. In [40], Transformer was introduced into a CNN-based fusion framework to improve the global dependencies of images. In their network, the CNN module composed of a structure branch and detail branch was utilized for shallow feature extraction, while the Transformer module was designed for the exploration of long-range dependencies.

However, most of these Transformer-based fusion methods simply combine the CNN and Transformer in a cascade manner to successively extract the local and global features. Moreover, they ignore the representation of multiscale features, which inevitably weakens significant information for fusion. Unlike these methods, we design a local–global parallel fusion network to adequately extract significant features from source images. In the proposed network, we introduce the Res2Net as a multiscale convolutional block into the local feature extraction module to exploit features at different scales. In addition, since the original vision Transformer can only output single-scale features, the multiscale Transformer is devised in our network to acquire coarse-to-fine semantic features.

## 3. Methods

In this section, a thorough introduction to the proposed fusion network RMTFuse is provided. Firstly, an overall framework of RMTFuse is briefly introduced. Then, we emphatically describe the fusion network. Finally, we present the hybrid loss function and its mathematical expression.

### 3.1. Overall Framework

**Figure 2:**
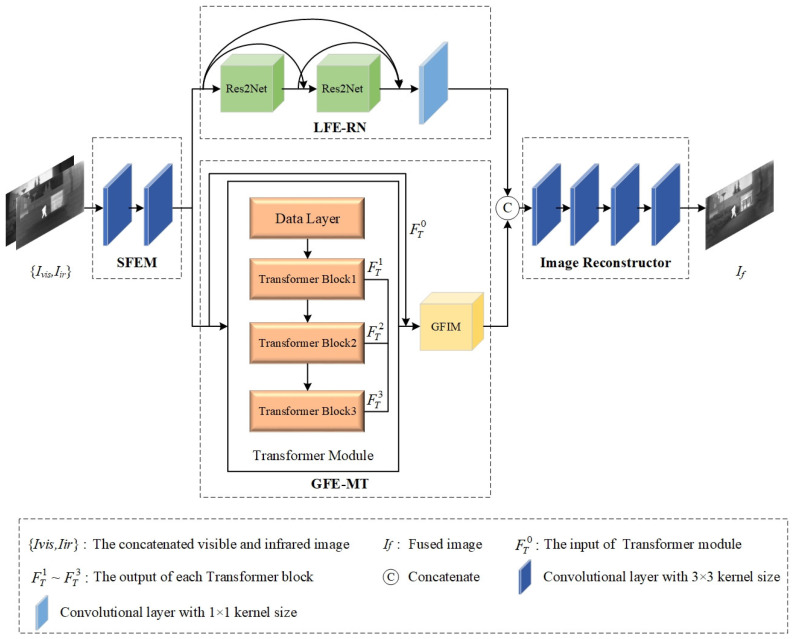
Overview of the proposed RMTFuse.

The proposed network is end-to-end and can be classified into two modules: the feature extraction module and the image reconstruction module. Firstly, the visible image $I_{vis}$ and the infrared image $I_{ir}$ are concatenated in the channel dimension. Then, they are sent to the shallow feature extraction module (SFEM) to obtain shallow features. Next, the mixed shallow features are input to a dual-stream structure, which comprises LFE-RN and GFE-MT, to acquire local and global complementary features simultaneously. Subsequently, the obtained local and global features are concatenated before being fed into the image reconstruction module to achieve feature reconstruction. Finally, we are able to obtain the fused result $I_{f}$. The overview of the proposed RMTFuse is shown in Figure 2. For convenience, Table 1 lists some abbreviations utilized in this section.

### 3.2. Network Architecture

As exhibited in Figure 2, the two-channel map $\left\{I_{vis},I_{ir}\right\}$ obtained by concatenating visible and infrared images is first sent to the SFEM which comprises two successive convolutional layers to extract shallow feature $F_{S}^{O}$. Each convolutional layer has a kernel size of 3 × 3, and LReLU is adopted as the activation function. Subsequently, local and global features are obtained by using parallel modules, LFE-RN and GFE-MT, respectively. In the end, the obtained feature maps are concatenated and transmitted to the image reconstructor to generate the final result. We will describe the LFE-RN and GFE-MT together with the image reconstructor in detail.

#### 3.2.1. Local Feature Extraction Module Based on Res2Net

To enable the network to learn texture details at different scales, we introduce the Res2Net blocks into the local feature extraction module. In Figure 2, it can be observed that LFE-RN deploys two Res2Net blocks and an ordinary convolutional layer. The Res2Net blocks represent the fine-grained multiscale features by aggregating multiple receptive fields hierarchically. Meanwhile, dense connections are adopted to achieve feature reuse, effectively retain multiscale detailed information, and improve fusion performance. Concretely, $F_{S}^{O}$ is sent to the first Res2Net block, which is expressed as

(1) $$F_{R1}^{O}={RB}_{1}(F_{S}^{O})$$

where ${RB}_{1}(\cdot )$ and $F_{R1}^{O}$ denote the first Res2Net block and the output intermediate features, respectively. Then,$\;F_{R1}^{O}$ and $F_{S}^{O}$ are concatenated and fed into the second block, which can be represented as

(2) $$F_{R2}^{O}={RB}_{2}(C(F_{S}^{O},F_{R1}^{O}))$$

where ${RB}_{2}(\cdot )$ indicates the second Res2Net block, C(·) indicates the concatenation conducted on the channel dimension, and $F_{R2}^{O}$ represents the obtained features extracted from the second Res2Net block. Subsequently, $F_{R2}^{O}$, $F_{R1}^{O}$, and $F_{S}^{O}$ are concatenated and sent to the last convolutional layer, which can be formulated as

(3) $$F_{LFE-RN}^{O}={Conv}_{1\times 1}(C(F_{S}^{O},F_{R1}^{O},F_{R2}^{O}))$$

where ${Conv}_{1\times 1}(\cdot )$ is an ordinary convolutional layer with kernel size 1 × 1 and LReLU activation function. It is deployed to eliminate channel dimension differences. $F_{LFE-RN}^{O}$ is the final result of LFE-RN. Table 2 shows the parameter settings of LFE-RN. *H* and *W* represent the height and width of input images.

#### 3.2.2. Global Feature Extraction Module Based on Multiscale Transformer

Due to the fact that the fusion performance will degrade without global context information, GFE-MT is devised for constructing long-range relationships. The design of GFE-MT is based on [41], as depicted in Figure 2. It includes two parts: the Transformer module and GFIM.

(1) Transformer Module

The fusion network can generate favorable fused results by introducing a primitive ViT module only. However, its fusion performance may be affected in the absence of global multiscale information. Therefore, we design a multiscale vision Transformer for global feature preservation across different scales. The Transformer module is made up of three different Transformer blocks with varied scales. To be exact, the overlapped patch merging module in [42] is employed within the original ViT model to acquire a hierarchical feature map, which has a resolution of $\frac{H}{2^{i+1}}\times \frac{W}{2^{i+1}}$. H×W means the size of input feature map, and $i\in \left\{1,2,3\right\}$. Moreover, the operation of overlapped patch merging can make the previously uncorrelated image block sequences correlative by setting *K* = 7, *S* = 4, *P* = 3, and *K* = 3, *S* = 2, *P* = 1, where *K*, *S*, and *P* mean convolutional kernel size, convolutional stride, and padding size. Table 3 illustrates the parameter settings of the Transformer module. ${TB}_{i}$(*i* = 1, 2, 3) represents the Transformer blocks. O-C symbolizes the number of output channels. E-D stands for the embedding dimensions. N-B denotes the number of Transformer encoders used within a Transformer block.

**Figure 3:**
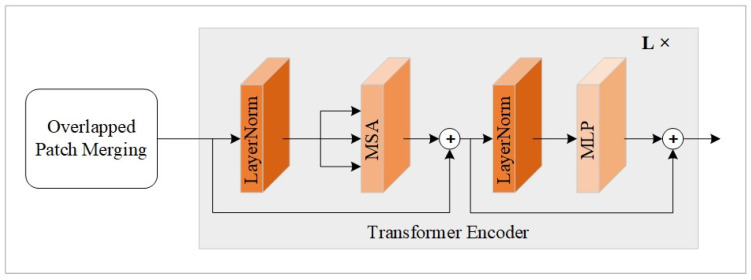
Detailed architecture of Transformer block.

The detailed architecture of the Transformer block adopted in our module is depicted in Figure 3. The obtained features will be fed into the subsequent Transformer encoder after the overlapped patch merging operation. The employed encoder comprises two LayerNorm (LN) units, multi-head self-attention (MSA), and a multi-layer perceptron (MLP). In addition, it has two residual connection operations, and the exact process can be formulated as follows:

(4) $$F_{RC}^{1}=MSA(LN(F_{T}^{I}))+F_{T}^{I}$$

(5) $$F_{RC}^{2}=MLP(LN(F_{RC}^{1}))+F_{RC}^{1}$$

where $F_{T}^{I}$ denotes the input of Transformer encoder. $F_{RC}^{1}$ and $F_{RC}^{2}$ represent the corresponding output results of the first and second residual connection operations.

(2) Global Feature Integration Module

**Figure 4:**
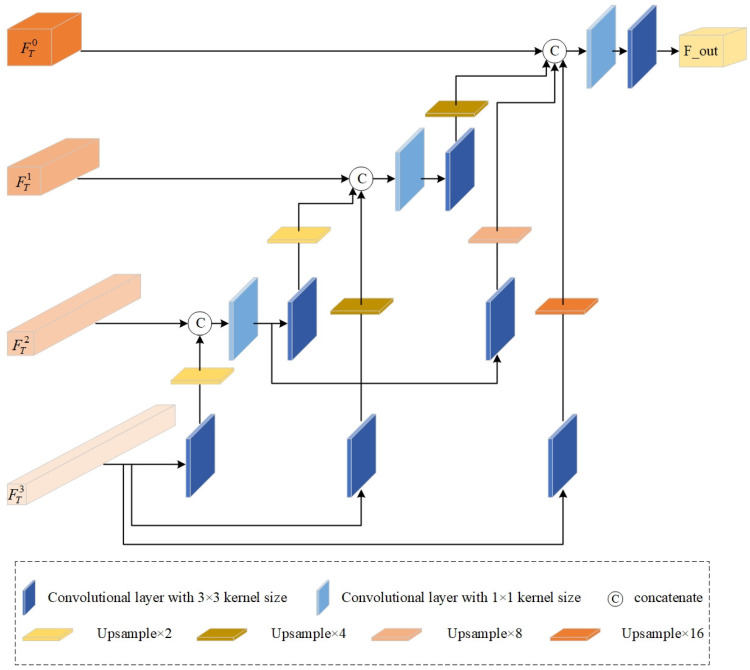
Detailed architecture of GFIM.

We can obtain hierarchical features through the multiscale Transformer module, from coarse to fine-grained semantic features. Therefore, our goal is to determine how to effectively integrate these features and strengthen the feature representations. To this end, we propose the GFIM which adopts convolution operation, upsampling, and channel connection to gradually recover the spatial size of input feature maps and merge channels. According to Figure 4, the core idea of GFIM is to cascade high-level scale features to low-level scale ones. In GFIM, $F_{T}^{i}$ represents the input tensor with a resolution of $\frac{H}{2^{i+1}}\times \frac{W}{2^{i+1}}\times C$, where $i\in \left\{1,2,3\right\}$, $C\in \left\{32,64,128\right\}$, and $F_{T}^{0}$ is the input of the Transformer module. We define ${Conv}_{3\times 3}(\cdot )$ as a convolutional block with 3 × 3 kernel size, followed by the LReLU activation function. Firstly, the fine-grained features $F_{T}^{3}$ derived from the third Transformer block are fed into convolutional blocks ${Conv}_{3\times 3}^{3}(\cdot )$, ${Conv}_{3\times 3}^{2}(\cdot )$, and ${Conv}_{3\times 3}^{1}(\cdot )$ via element-level projection mapping to acquire features $F_{33}^{\prime }$, $F_{32}^{\prime }$, and $F_{31}^{\prime }$. Obviously, although those obtained feature maps are consistent with the lower-level feature information $F_{T}^{2}$, $F_{T}^{1}$, and $F_{T}^{0}$ in the channel dimension, their spatial sizes do not coincide. Therefore, bicubic interpolation is employed to upsample feature maps $F_{33}^{\prime }$, $F_{32}^{\prime }$, and $F_{31}^{\prime }$ to ensure they have the same spatial size as $F_{T}^{2}$, $F_{T}^{1}$, and $F_{T}^{0}$, respectively. The specific calculated process is denoted as

(6) $$F_{3j}^{\prime \prime }=UP({Conv}_{3\times 3}^{j}\left(F_{T}^{3}\right))$$

where $j\in \left\{1,2,3\right\}$, and $F_{3j}^{\prime \prime }$ is the feature map acquired by the upsampling operation. UP(·) denotes the upsampling operation.

The obtained $F_{33}^{\prime \prime }$ and feature maps $F_{T}^{2}$ are then concatenated for the purpose of merging global information of $F_{T}^{3}$ and $F_{T}^{2}$. The concatenated features are first projected and mapped by a convolution block ${Conv}_{1\times 1}^{2}(\cdot )$ with kernel size 1 × 1 and LReLU activation function. To interact with lower-scale feature $F_{T}^{1}$ and $F_{T}^{0}$, the feature maps $F_{T}^{2\sim }$ obtained by the above projection mapping are then sent to two convolutional blocks ${Conv}_{3\times 3}^{5}(\cdot )$ and ${Conv}_{3\times 3}^{4}(\cdot )$ and subsequent upsampling blocks to obtain feature maps $F_{22}^{\prime \prime }$ and $F_{21}^{\prime \prime }$ which are consistent with $F_{T}^{1}$ and $F_{T}^{0}$ in channel dimension and spatial size, respectively. The specific process is formulated as

(7) $$F_{2j}^{\prime \prime }=UP({Conv}_{3\times 3}^{j+3}\left({Conv}_{1\times 1}^{2}(C(F_{T}^{2},F_{33}^{\prime \prime })\right)))$$

where $j\in \left\{1,2\right\}$ and C(·) implies the operation of concatenation.

To effectively integrate shallow and deep feature information, the obtained feature maps $F_{32}^{\prime \prime }$ and $F_{22}^{\prime \prime }$ are concatenated with input tensor $F_{T}^{1}$. Subsequently, the concatenated features $F_{T}^{1\sim }$ pass through convolutional blocks ${Conv}_{1\times 1}^{1}(\cdot )$ and ${Conv}_{3\times 3}^{6}(\cdot )$ and an upsampling block to obtain feature maps $F_{11}^{\prime \prime }$ that could interact with input $F_{T}^{0}$. Then, we concatenate $F_{31}^{\prime \prime }$, $F_{21}^{\prime \prime }$, $F_{11}^{\prime \prime }$, and $F_{T}^{0}$ and send them to convolutional blocks ${Conv}_{1\times 1}^{0}(\cdot )$ and ${Conv}_{3\times 3}^{7}(\cdot )$ successively to obtain the final output feature maps $F_{GFE-MT}^{O}$. The whole calculation process is denoted as follows:

(8) $$F_{11}^{\prime \prime }=UP({Conv}_{3\times 3}^{6}({Conv}_{1\times 1}^{1}(C(F_{32}^{\prime \prime },F_{22}^{\prime \prime },F_{T}^{1}))))$$

(9) $$F_{GFE-MT}^{O}={Conv}_{3\times 3}^{7}({Conv}_{1\times 1}^{0}(C(F_{31}^{\prime \prime },F_{21}^{\prime \prime },F_{11}^{\prime \prime },F_{T}^{0})))$$

#### 3.2.3. Image Reconstructor

In our network, the obtained local features $F_{LFE-RN}^{O}$ and global features $F_{GFE-MT}^{O}$ are then integrated via a concatenation strategy, and these aggregated feature maps are sent to the image reconstructor to generate the fused result. As shown in Figure 2, the image reconstructor consists of four successive convolutional layers with 3 × 3 kernel size and stride = 1.

### 3.3. Loss Function

As the proposed RMTFuse adopts unsupervised learning, the design of the loss function in the model is essential to the quality of fused results. In order to constrain the generated fusion images to capture additional complementary features from inputs, for example, the abundant texture details and significant infrared targets, we devise a hybrid loss function. It comprises per-pixel loss $L_{pixel}$ and perceptual loss $L_{per}$, and the expression is as follows:

(10) $$L_{total}=L_{pixel}+\alpha L_{per}$$

where α is a weight-off factor for balancing the total loss function.

#### 3.3.1. Per-Pixel Loss

This part of the loss includes $L_{ssim}$ and $L_{TV}$, which can be formulated as

(11) $$L_{pixel}=L_{ssim}+\beta L_{TV}$$

where $L_{ssim}$ and $L_{TV}$ represent SSIM loss and total variation loss, respectively. β is a weighting factor used to control the contributions of each term.

The structural similarity (SSIM) index can reflect distortion from different perspectives, such as brightness, contrast, and structure [43]. With the aim of maintaining more structure information from input images, we introduce SSIM loss to constrain the structural similarity of output and input images. It is computed as

(12) $$L_{ssim}=1-SSIM(I_{f},I_{s})$$

where $I_{f}$ is the output and $I_{s}$ denotes the source images. SSIM(·) represents the structural similarity measurement.

To better retain the gradient information in original images and eliminate the noise of generated results, we employed the total variation (TV) loss introduced in VIF-Net [44]. It is formulated as

(13) $$T\left(p,q\right)=I_{f}\left(p,q\right)-I_{s}(p,q)$$

(14) $$L_{TV}=\underset{p,q}{\sum }\left({\left\|T\left(p,q+1\right)-T\left(p,q\right)\right\|}_{2}+{\left\|T\left(p+1,q\right)-T\left(p,q\right)\right\|}_{2}\right)$$

where T(·)  means the difference between the source images and the fused image. ${\left\|\cdot \right\|}_{2}$ represents the $l_{2}$ norm, while p and q denote the horizontal and vertical coordinates of the image pixels, respectively.

#### 3.3.2. Perceptual Loss

It challenging to acquire the perceptual difference between source images and generated images in the image fusion field by only per-pixel loss [45]. For instance, two identical images differing from each other by just a few pixels may be quite different when measured in terms of per-pixel loss, despite their perceptual similarity. To this end, we introduce perceptual loss into hybrid loss for retaining high similarity between merged images and original images, so as to further improve fusion performance. It is denoted as

(15) $$L_{per}=\underset{k=2,4,6}{\sum }{\left\|\varphi^{k}(I_{f})-\varphi^{k}(I_{s})\right\|}_{1}$$

where $\varphi^{k}$ indicates the *k*-th layer feature map obtained from the pre-trained VGG-16 model [46]. ${\left\|\cdot \right\|}_{1}$ denotes the $l_{1}$ norm.

## 4. Experiments and Discussion

In this section, we first present the training details of our RMTFuse. Then, we provide the comparison algorithms and objective indices. Next, ablation experiments are performed to validate the effectiveness of our network structure. Afterward, the comparative and generalization experiments are presented to illustrate the superiority of RMTFuse. We also provide the computational complexity analysis. In the end, object detection experiments are conducted to assess different fusion models in the context of computer visual challenges.

### 4.1. Training Details

In this work, we choose 36 pairs of corresponding infrared and visible images from the TNO [47] benchmark as the training set. In order to create enough samples for training our model, original image pairs are cropped into patches of size 256×256 by setting 12 for the overlapping cropping step. Thus, approximately 20,000 image patches are collected and converted into grayscale images. We employ the Adam optimizer to update weights and set the learning rate to 0.0001 during the process of training. Meanwhile, the batch size and epoch are established at 8 and 30, respectively. The weight parameters, α and β, are set to 0.001. Moreover, our model is built on the PyTorch framework, and all experiments are implemented on the NVIDIA GeForce RTX 4090 GPU (NVIDIA Corporation, Santa Clara, CA, USA).

### 4.2. Comparison Methods and Objective Indices

In our experiments, two publicly accessible datasets, i.e., TNO [47] and RoadScene [24], are used to comprehensively estimate the fusion quality of our RMTFuse. Nine state-of-the-art image fusion methods, including BF [14], FusionGAN [20], DenseFuse [27], GANMcC [31], LRRNet [48], YDTR [49], SwinFusion [38], DATFuse [39], and CMTFusion [50], are picked for performance comparison. All source codes of the comparison methods above are accessible to the public, and we configure the parameters following the recommendations of the corresponding papers.

For quantitative evaluation, eight widely used metrics are deployed for assessing the fusion results objectively, which include standard deviation (SD) [51], visual information fidelity (VIF) [52], entropy (EN) [53], gradient-based fusion performance (*Q*_abf_) [54], spatial frequency (SF) [55], modified fusion measure (*N*_abf_) [56], multiscale SSIM (MS-SSIM) [57], and feature mutual information with pixel (FMI_pixel_) [58]. SD reflects the distribution and contrast of fusion results in a statistical way. VIF computes the distortion of the image to measure the information fidelity in fusion results in the view of the human visual system. EN indicates the amount of information that an image contains. *Q*_abf_ aims to measure the level of edge information transmitted from original images to the final generated result. SF evaluates the change rate of the image gray level and reveals the textural characteristics of an image. *N*_abf_ aims to compute noise and artifacts added to the generated results. MS-SSIM measures the similarity between the original images and the fusion image on different levels of scale. FMI_pixel_ calculates mutual information of features from pixel features. Moreover, except for *N*_abf_, the higher the value of the evaluation index, the more effective the fusion network is.

### 4.3. Ablation Experiments

Owing to the introduction of LFE-RN and GFE-MT in the proposed RMTFuse, it is equipped with the ability to extract local and global complementary features from source images adequately, thereby generating informative fused results. To verify the effects of LFE-RN and GFE-MT, 25 image pairs from the TNO dataset are selected to perform ablation studies. To validate the necessity of LFE-RN, we replace the Res2Net blocks with normal convolution layers, named w/o RN. To illustrate the significance of GFE-MT, we remove the GFE-MT from the whole architecture, called w/o GFE-MT. Similarly, the original ViT module is employed to replace the multiscale Transformer module to explore the impacts of the proposed multiscale vision Transformer structure in GFE-MT, termed w/o MT.

**Figure 5:**
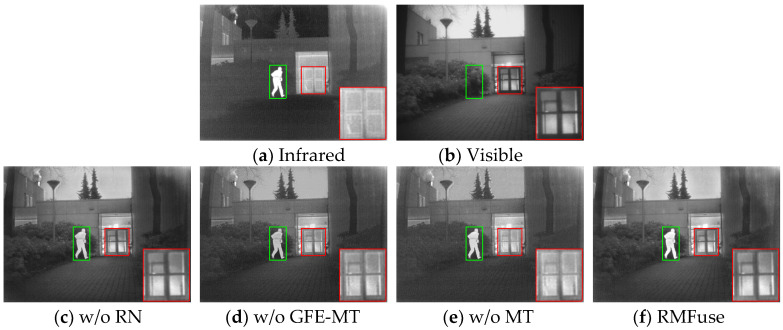
Representative results of ablation experiment on different structures.

The ablation experiment results on the TNO dataset of different structures are depicted in Figure 5. Two local areas of each fusion result are marked, and one of them is zoomed in as a close-up for better observation. Clearly, the fusion result of w/o GFE-MT suffers from insignificant infrared targets as a result of lacking the global feature extraction ability. Moreover, some important detailed textures are lost, leading to unclear fusion results. With the addition of the original ViT module, more prominent features of the source images are maintained, leading to a brighter target person in the fused result of w/o MT. However, the overall scene of w/o MT is drowned in darkness, which is unable to bring a pleasant visual experience. In contrast, the fused result of w/o RN alleviates the darkened result to some extent, but the thermal target is still contaminated by the visible image compared with our fused image. With the introduction of Res2Net and multiscale Transformer module, our result achieves the best fusion performance, which not only maintains the prominent information in infrared images, but also exhibits excellent ability in preserving the texture details, such as the clear door frame and floor. From the ablation experiment results above, it can be seen that adding the multiscale Transformer module can enhance the long-range dependency modeling, which enables the proposed model to make the best of local and global information. In addition, the Res2Net block in the proposed network also facilitates the preservation of useful complementary information in source images.

The objective comparisons of our RMTFuse with other network structures are depicted in Table 4. The top-performing result for each evaluation metric is marked in bold, while the second-best result is underlined. It is clear that the complete network structure displays optimal performance over all metrics, which is consistent with the subjective evaluation results. These experimental results all show that our proposed network structure is effective and reasonable.

### 4.4. Comparative Experiments

**Figure 6:**
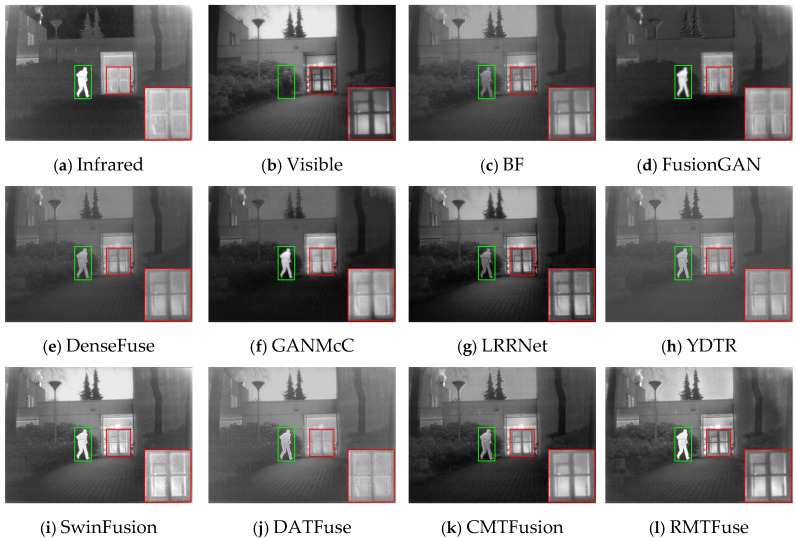
Subjective results of RMTFuse with 9 different methods on *Kaptien_1123*.

**Figure 7:**
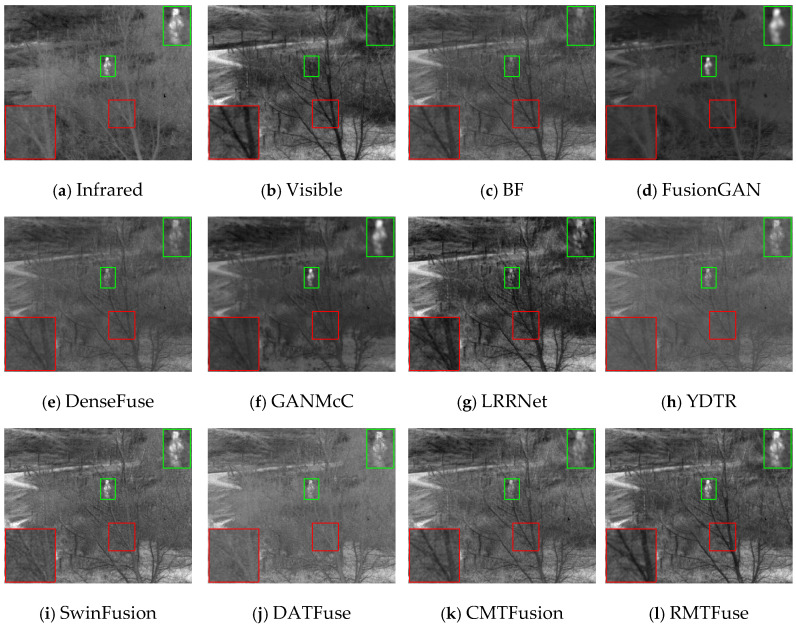
Subjective results of RMTFuse with 9 different methods on *Sandpath_18*.

**Figure 8:**
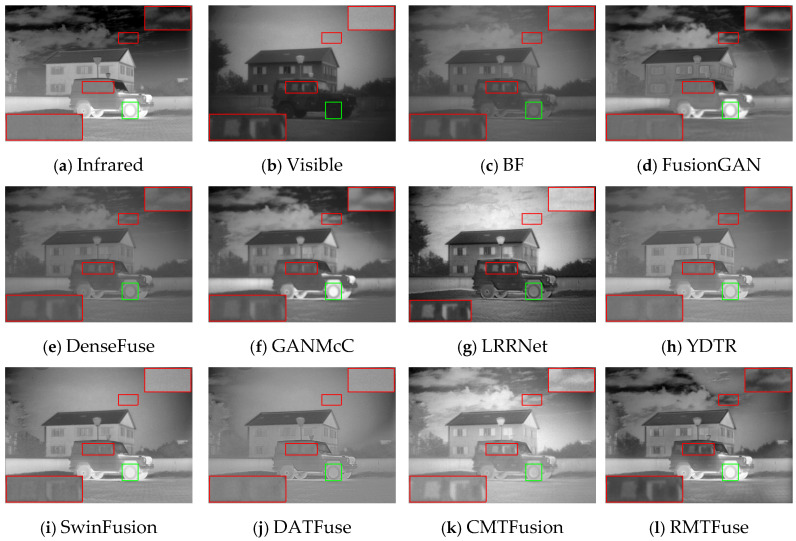
Subjective results of RMTFuse with 9 different methods on *Marne_04*.

To intuitively illustrate the strength of our model, we pick out three representative scenes from the TNO dataset: (1) *Kaptien_1123*; (2) *Sandpath_18*; (3) *Marne_04*. The three sets of infrared and visible images along with their corresponding fusion images obtained by different methods under the same environment are shown in Figure 6, Figure 7 and Figure 8. And we use green boxes and red boxes to mark salient objects and texture details, respectively, to display the fusion difference clearly. Some marked areas are then magnified and positioned in the corners. Despite the favorable fused images achieved by nine comparison algorithms, there are still some limitations compared with RMTFuse. Specifically, BF, DenseFuse, YDTR, and DATFuse can preserve part of the texture details, but the intensity information of prominent objects is weakened to different degrees, such as the targets in Figure 6 and Figure 7 and the tires and engine of the jeep in Figure 8. Moreover, the merged images generated by these algorithms have lower contrast. Similarly, the fused results of LRRNet exhibit low brightness of infrared targets, which is unfavorable for object tracking and detection tasks. Although FusionGAN and GANMcC are able to successfully highlight the thermal targets, the obtained fused results are quite fuzzy, which leads to the loss of substantial scene information, such as the floor and sky in Figure 6. Similar phenomena can be observed in Figure 7 and Figure 8. In contrast, the fusion results obtained by CMTFusion have good contrast and successfully preserve the meaningful information of original images. Unfortunately, the infrared target is still weakened to a certain extent. It is notable that the fusion performance of SwinFusion outperforms the aforementioned approaches; however, the ability of our RMTFuse to preserve texture details is better. As presented in Figure 6 and Figure 8, the proposed RMTFuse effectively maintains the high-contrast areas in infrared images and clearly shows the door frame, the jeep windows, and the edge of clouds. Similarly, in Figure 7, the tree branches are apparent in our fusion result, which is unmatched by other algorithms. Overall, our RMTFuse provides the best visual experience. Moreover, the fusion results generated by our network contain both salient infrared targets and rich texture information.

In order to further demonstrate the excellent performance of RMTFuse, 25 image pairs from the TNO dataset are selected to perform the objective assessment. The mean values of eight different assessment metrics calculated from these images generated by the aforementioned fusion approaches are listed in Table 5. The optimal metric value is highlighted in bold, while the second-best metric value is underlined. It is clear that our RMTFuse performs the best in all metrics. The optimal VIF and SD illustrate that our fused results provide satisfying visual effects and high contrast. The highest EN means scene information is effectively maintained in the fused images. Moreover, RMTFuse obtains the highest values in SF, MS-SSIM, and *Q*_abf_, indicating that we can preserve more source image details, structural information, and edge information. The best FMI_pixel_ metric means the proposed RMTFuse conserves a great deal of feature information transmitted from the original image. Furthermore, the lower *N*_abf_ reveals that our results contain less noise and artifacts. Overall, our method delivers the optimal fusion performance. Undoubtedly, this result is in line with the subjective assessment.

### 4.5. Generalization Experiments

**Figure 9:**
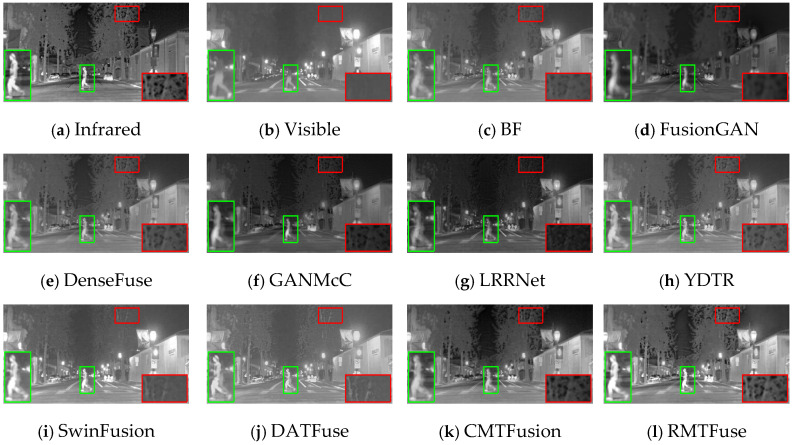
Subjective results of RMTFuse with 9 different methods on *FLIR_08999*.

**Figure 10:**
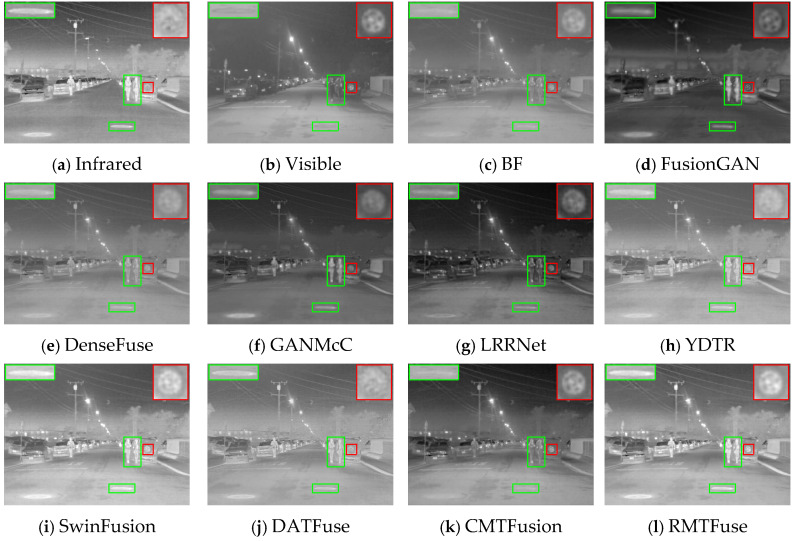
Subjective results of RMTFuse with 9 different methods on *FLIR_07732*.

**Figure 11:**
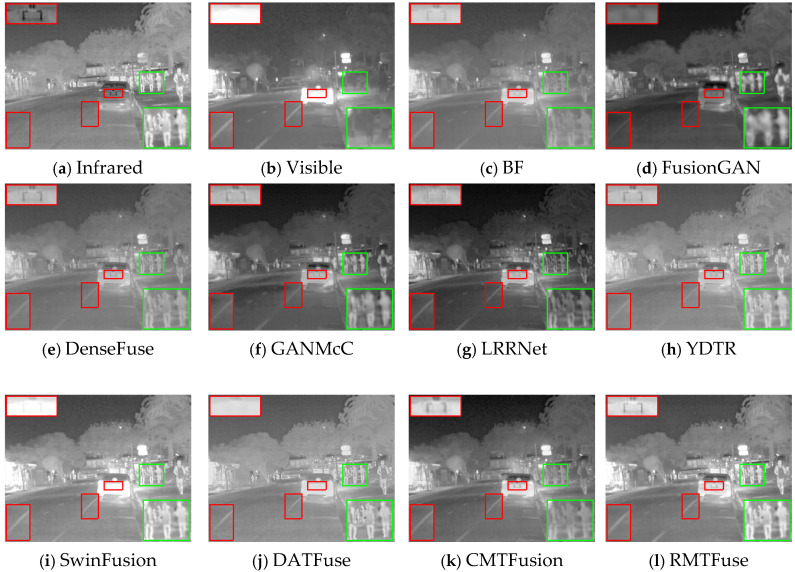
Subjective results of RMTFuse with 9 different methods on *FLIR_08202*.

The quality of generalization experiments is a significant factor in evaluating data-driven algorithms. Hence, we carry out comparison experiments on the RoadScene dataset for the purpose of verifying the generalization capability of the proposed RMTFuse. Notably, the proposed model of RMTFuse is trained on the TNO dataset and directly tested on the RoadScene dataset. For subjective evaluation, three representative scenes are selected to present the fused results, as shown in Figure 9, Figure 10 and Figure 11. It can be observed that the traditional method BF loses some local details and thermal information, such as the pedestrians in every scene, the tree branches in Figure 9, and the manhole covers in Figure 10. Similarly, the thermal radiation information is unremarkable in the generated results of DenseFuse and CMTFusion. Although these two methods can successfully preserve some texture details, they cannot learn salient information in infrared images. The fused images of FusionGAN are relatively blurred and lose lots of scene textures. Despite the fact that GANMcC and YDTR have more texture information, compared with our method, the obtained texture details are still limited, such as the tire details in the car rear in Figure 10. As for the LRRNet method, it fails to retain infrared thermal information sufficiently, for example, the significant targets in each scene. Obviously, our RMTFuse performs well in retaining salient objects and texture information simultaneously. As shown in Figure 11, the pedestrian is salient and the street line is clear in the fused result of RMTFuse. Unfortunately, there is a bias in the integration of the brightness information in both SwinFusion and DATFuse, resulting in an overexposure scene and missing the boundary information and detailed textures in the car rear. The same result can also be observed in other scenes.

We randomly pick 40 image pairs from the RoadScene dataset to evaluate objectively. Table 6 displays the average values of RMTFuse with the other nine comparative fusion approaches on different assessment metrics. It is evident that our RMTFuse performs best on six metrics, which include SD, VIF, *Q*_abf_, SF, *N*_abf_, and MS-SSIM. This reveals that our fused results not only preserve texture details and abundant information, but also have the best visual perception and the least amount of noise. Furthermore, RMTFuse ranks second in EN and FMI_pixel_ metrics, which means that the fusion results generated by RMTFuse contain abundant scene information and feature information.

### 4.6. Computational Complexity Analysis

To evaluate the computation complexity of the proposed RMTFuse, we present the average running time of different methods on TNO and RoadScene datasets in Table 7. The traditional method BF is implemented on a computer platform with an i9-13900KS CPU, and deep-learning-based methods are conducted on an NVIDIA GeForce RTX 4090 GPU. As shown in Table 7, our RMTFuse ranks moderate among all the fusion methods owing to the introduction of the multiscale Transformer module. However, it achieves better fusion performance compared with other methods. In general, the proposed RMTFuse remains competitive.

### 4.7. Detection Performance

To validate the effectiveness of our RMTFuse in facilitating computer vision tasks, we evaluate the fused results generated by different methods using a prevailing object detection method. Specifically, the YOLOX detector [59] is utilized for detection. And we randomly select 52 image pairs from the MFNet [3] dataset for evaluation, including both daytime and nighttime images.

**Figure 12:**
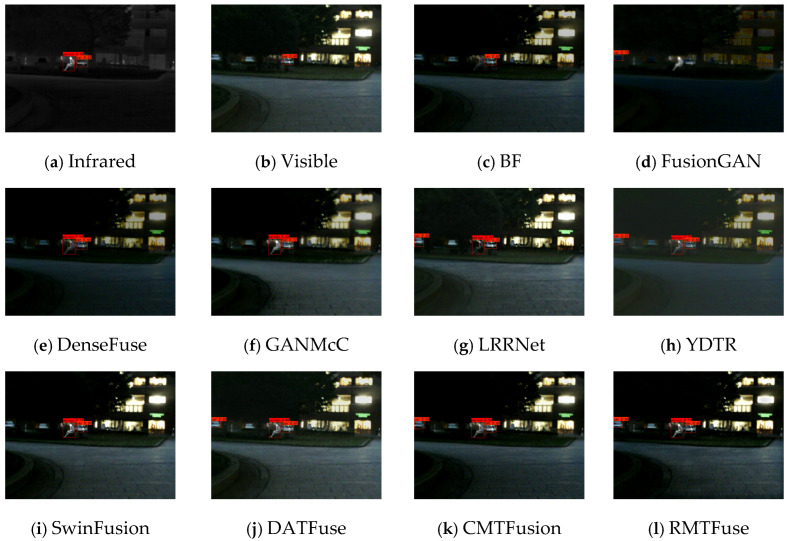
Object detection results on scene *00008N*.

**Figure 13:**
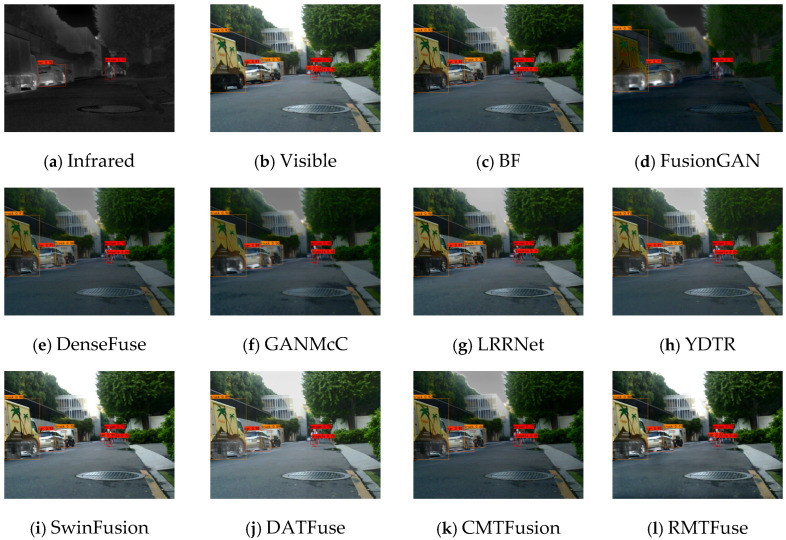
Object detection results on scene *00315D*.

Figure 12 and Figure 13 show some typical objection detection results of the source images and different fused results. In the *00008N* scene, BF is unable to maintain the thermal target, while FusionGAN cannot keep the sharpened edges of the pedestrian target. Consequently, the detector cannot detect the pedestrian from the fusion results. It can be observed that only LRRNet, YDTR, DATFuse, CMTFusion, and our RMTFuse detect both people and two cars. However, due to the interference of negative information, LRRNet and CMTFusion weaken the significant targets, resulting in low confidence in pedestrian targets. In addition, the YDTR and DATFuse fail to preserve the detailed textures in source images, which leads to lower confidence in detecting cars than our RMTFuse. A similar phenomenon can be seen in the *00315D* scenario. In the *00315D* scene, the visible image has lower confidence for pedestrian detection than the infrared image as a result of illumination factors. SwinFusion and the proposed RMTFuse accurately detect all targets in the scene, while the remaining methods fail to detect the car in the distance. Overall, our method fully integrates thermal targets and texture details from source images and has a confidence level closer to the source images over all detected objects. This indicates that RMTFuse is more conducive to computer vision tasks.

To further measure the detection performance of different approaches, we utilize the mean average precision (mAP) to conduct a quantitative assessment. Table 8 shows the assessment results. mAP@0.5 and mAP@0.9 imply the mAP values when the IoU threshold is 0.5 and 0.9, respectively. The closer the mAP value is to 1, the higher the quality of object detection. Obviously, the proposed RMTFuse achieved the highest average detection accuracy at both thresholds, which reveals the optimal detection performance. And we can also observe that, in comparison to other fusion algorithms, our RMTFuse has a higher accuracy of detection on the car. This indicates that our method is able to better maintain the valuable information in infrared and visible images. In brief, our RMTFuse excels in the image fusion task, which in turn helps to improve object detection accuracy.

## 5. Conclusions

In this study, we develop a novel end-to-end image fusion model for infrared and visible images, named RMTFuse. The proposed network utilizes the strengths of the Res2Net block and multiscale Transformer structure to achieve the extraction of local and global complementary features. In our network, we devise a local feature extraction module based on Res2Net (LFE-RN) to capture multiscale local features and adopt dense connections in the module to achieve feature reuse. To realize long-range dependency modeling and further expand the receptive field, we develop a global feature extraction module based on multiscale Transformer (GFE-MT), in which a global feature integration module (GFIM) is devised for aggregating feature information at different scales. It is worth remarking that LFE-RN and GFE-MT are ordered in parallel, allowing more efficient utilization of local detail information and global contextual features. Moreover, we utilize the pre-trained VGG-16 network for extracting features at different levels to compute the loss. Massive experiments on the TNO and RoadScene datasets illustrate that our proposed RMTFuse surpasses current state-of-the-art approaches in terms of both subjective effects and objective evaluations. Furthermore, ablation experiments are performed to verify the effects of different module designs employed in the proposed network. And the extended experiments on object detection also reveal the advantages of RMTFuse for computer vision tasks. In the future, we expect to further study how to integrate the image alignment task with the image fusion task organically to realize a unified model for unaligned images. We will also generalize the algorithm to different image fusion fields, for example, multi-focus image fusion, medical image fusion, and so on.

## Figures and Tables

**Table 1:** List of some abbreviations.

Abbreviation	Description
SFEM	Shallow feature extraction module
LFE-RN	Local feature extraction module based on Res2Net
GFE-MT	Global feature extraction module based on multiscale Transformer
GFIM	Global feature integration module
MSA	Multi-head self-attention
MLP	Multi-layer perceptron

**Table 2:** Detailed parameter design of LFE-RN.

Layer	Output Size	Input Channel	Output Channel	Kerner Size	Stride	Activation
RB1	H×W	16	16	---	---	---
RB2	H×W	32	16	---	---	---
${Conv}_{1}$	H×W	48	32	1	1	LReLU

**Table 3:** Detailed parameter design of Transformer module.

	Output Size	Layer	O-C	K	S	P	E-D	N-B
TB1	$\frac{H}{4}\times \frac{W}{4}$	Overlapped Patch Merging	32	7	4	3	---	----
		Transformer Encoder	---	---	---	---	32	3
TB2	$\frac{H}{8}\times \frac{W}{8}$	Overlapped Patch Merging	64	3	2	1	---	---
		Transformer Encoder	---	---	---	---	64	4
TB3	$\frac{H}{16}\times \frac{W}{16}$	Overlapped Patch Merging	128	3	2	1	---	---
		Transformer Encoder	---	---	---	---	128	6

**Table 4:** Objective comparison results of different structures on TNO dataset.

Method	SD	VIF	EN	*Q*_abf_	SF	*N*_abf_	MS-SSIM	FMI_pixel_
w/o RN	39.3140	0.7510	7.0783	0.5486	11.3897	0.0277	0.9340	0.9045
w/o GFE-MT	34.3967	0.6418	6.8303	0.4948	11.2254	0.0311	0.9291	0.9029
w/o MT	38.3660	0.7093	7.0015	0.5338	11.2353	0.0259	0.9293	0.9040
RMTFuse	**40.5153**	**0.7713**	**7.0949**	**0.5621**	**11.9282**	**0.0255**	**0.9342**	**0.9085**

**Table 5:** Objective comparison results on TNO dataset.

Method	SD	VIF	EN	*Q*_abf_	SF	*N*_abf_	MS-SSIM	FMI_pixel_
BF	29.3930	0.6704	6.5879	0.4422	8.2445	0.0993	0.8677	0.9080
FusionGAN	29.1010	0.4183	6.4850	0.2245	6.2654	0.0772	0.7288	0.8858
DenseFuse	25.2951	0.5869	6.4495	0.3521	6.8819	0.0830	0.8739	0.9048
GANMcC	31.6695	0.5246	6.6922	0.2770	6.3030	0.0687	0.8557	0.8964
LRRNet	39.2691	0.5604	6.9719	0.3626	9.6324	0.0562	0.8514	0.8921
YDTR	26.9168	0.6130	6.4626	0.3937	7.9214	0.0586	0.8505	0.8988
SwinFusion	39.4348	0.7592	6.9321	0.5321	11.3832	0.0359	0.8931	0.9059
DATFuse	28.0275	0.6929	6.5187	0.4966	9.8825	0.0435	0.8054	0.8740
CMTFusion	36.0810	0.6755	6.9769	0.4890	10.6160	0.0527	0.9233	0.9037
RMTFuse	**40.5153**	**0.7713**	**7.0949**	**0.5621**	**11.9282**	**0.0255**	**0.9342**	**0.9085**

**Table 6:** Objective comparison results on RoadScene dataset.

Method	SD	VIF	EN	*Q*_abf_	SF	*N*_abf_	MS-SSIM	FMI_pixel_
BF	30.4217	0.5909	6.7010	0.3348	7.7928	0.1841	0.7795	**0.8680**
FusionGAN	38.0279	0.3806	7.0412	0.2562	8.0454	0.1458	0.7562	0.8533
DenseFuse	31.7208	0.5791	6.8133	0.3928	8.3005	0.1470	0.8588	0.8641
GANMcC	41.7463	0.5135	7.1743	0.3473	8.6212	0.1291	0.8495	0.8560
LRRNet	41.9076	0.4855	7.1070	0.3403	11.8798	0.1054	0.7976	0.8511
YDTR	35.8128	0.5833	6.8862	0.4483	10.2754	0.0992	0.8622	0.8616
SwinFusion	44.6716	0.6288	6.9886	0.4676	11.7787	0.0742	0.8499	0.8593
DATFuse	32.3401	0.5970	6.7239	0.4871	11.4661	0.0834	0.7611	0.8546
CMTFusion	45.3994	0.6226	**7.3355**	0.4433	11.6916	0.0983	0.8919	0.8584
RMTFuse	**47.2999**	**0.6549**	7.3002	**0.5610**	**13.8569**	**0.0359**	**0.9339**	0.8654

**Table 7:** The average running time of different fusion methods on TNO and RoadScene datasets (unit: seconds).

Method	TNO	RoadScene
BF	1.5734	0.2803
FusionGAN	0.2686	0.2924
DenseFuse	0.1025	0.0521
GANMcC	0.4600	0.5215
LRRNet	0.1722	0.0918
YDTR	0.3961	0.1085
SwinFusion	1.8124	0.8726
DATFuse	0.0609	0.0377
CMTFusion	0.2208	0.1294
RMTFuse	0.2802	0.1072

**Table 8:** Object detection performance for infrared, visible, and different fused images.

Method	mAP@0.5			mAP@0.9		
	Person	Car	Avg.	Person	Car	Avg.
IR	0.7315	0.2547	0.4931	0.2657	0.2498	0.2578
VIS	0.5362	0.7330	0.6346	0.2103	**0.5049**	0.3576
BF	0.6227	0.6742	0.6485	0.2616	0.4381	0.3499
FusionGAN	0.5173	0.5335	0.5254	0.1166	0.3426	0.2296
DenseFuse	0.7250	0.6437	0.6844	0.2712	0.4279	0.3496
GANMcC	0.7114	0.6809	0.6962	0.2170	0.4142	0.3156
LRRNet	0.6884	0.7395	0.7140	0.2242	0.4581	0.3412
YDTR	0.7350	0.7070	0.7210	0.2668	0.4593	0.3631
SwinFusion	0.7195	0.7388	0.7292	0.2611	0.4267	0.3439
DATFuse	**0.7655**	0.7253	0.7454	**0.2777**	0.3853	0.3315
CMTFusion	0.6962	0.7012	0.6987	0.2665	0.4086	0.3376
RMTFuse	0.7365	**0.7549**	**0.7457**	0.2685	0.4698	**0.3692**

## Data Availability

Source of dataset in experimental analysis: https://figshare.com/articles/dataset/TNO_Image_Fusion_Dataset/1008029 (accessed on 10 June 2024), https://github.com/hanna-xu/RoadScene (accessed on 10 June 2024), and https://github.com/haqishen/MFNet-pytorch (accessed on 12 June 2024).
